# Case Report: Dupilumab induced sustained complete remission in a child with severe alopecia areata and markedly elevated IgE

**DOI:** 10.3389/fimmu.2026.1832906

**Published:** 2026-05-01

**Authors:** Zexin Zhao, Fan Yang, Liancheng Guan, Deping Luo, Hongxia Li

**Affiliations:** The Second Affiliated Hospital, Guizhou University of Traditional Chinese Medicine, Guiyang, China

**Keywords:** alopecia areata, atopic constitution, complete remission, dupilumab, high IgE, pediatric

## Abstract

**Background:**

Severe alopecia areata in children is difficult to treat, and conventional therapies often show limited efficacy and substantial adverse effects. Dupilumab has shown potential in patients with alopecia areata and an atopic background, and baseline IgE has been proposed as a predictor of response. However, long-term outcomes and maintenance strategies in children with very high IgE levels remain insufficiently described.

**Case presentation:**

We report a 7-year-old girl with severe alopecia areata (SALT score 95) and a disease d uration of more than 2 years who had responded poorly to previous glucocorticoid therapy. She had a history of atopic dermatitis (AD), and her serum total IgE level was 2300 IU/mL. She received dupilumab 300 mg subcutaneously every 4 weeks in combination with topical corticosteroids and, later, topical minoxidil. Hair regrowth was observed after 8 weeks. The SALT score decreased to 5 at week 30, and complete remission was achieved at week 48 (SALT score 0). Serum total IgE decreased in parallel to 330 IU/mL. After near-complete response, the dosing interval was successfully extended to 6 weeks, and efficacy was maintained through week 48. The only adverse event was a transient mild allergic reaction after the first injection.

**Conclusion:**

A progressive reduction in serum total IgE levels was observed in parallel with the patient’s clinical improvement. This case supports dupilumab as a potentially effective and well-tolerated option for severe pediatric alopecia areata in the setting of markedly elevated baseline IgE. It also suggests that dynamic monitoring of​ IgE levels may be clinically informative and that interval extension during maintenance therapy may be feasible after a robust response.

## Introduction

Alopecia areata is a common T-cell-mediated autoimmune, non-scarring hair loss disorder, with a global prevalence of approximately 0.2%; the prevalence in children is slightly higher than that in adults (1, 2). Severe alopecia areata in children affects not only appearance but also mental health and may cause anxiety, depression, and other psychological problems, thereby markedly reducing quality of life (3, 4). In addition, pediatric alopecia areata is often more difficult to treat than adult disease because of earlier onset, an immature immune system, which has been associated with a less favorable prognosis (1, 2),and the frequent coexistence of poor prognostic factors such as an atopic background. Conventional treatments, including systemic or topical corticosteroids and immunosuppressants, have limitations in both efficacy and safety and are not suitable for long-term use, especially in young children (5–8).

The conventional view is that Th1 responses are the main driver of alopecia areata. However, recent studies suggest that the Th2 pathway also plays an important role in some patients, particularly those with an atopic background. Supporting evidence includes the high comorbidity of alopecia areata with atopic diseases, upregulation of Th2-related cytokines such as IL-4 and IL-13, and elevated serum IgE levels (9, 10).

Dupilumab is a monoclonal antibody targeting IL-4Rα. By blocking IL-4/IL-13 signaling, it suppresses Th2-driven inflammation and was first approved for moderate-to-severe atopic dermatitis (11). A phase IIa clinical trial in adults with alopecia areata confirmed its efficacy and proposed baseline IgE ≥200 IU/mL as a predictor of treatment response (12). Several pediatric case reports and case series have also described its efficacy and safety in children (13–16). However, long-term follow-up data and information on maintenance strategies in children with very high IgE levels remain scarce.

Here, we report a child with severe alopecia areata, atopic dermatitis, and markedly elevated serum IgE who achieved complete remission after dupilumab treatment and maintained benefit after extension of the dosing interval. The case is notable for the extreme baseline IgE elevation, the parallel decline in IgE and SALT score, and the apparent success of individualized maintenance dosing.

## Case description

### De-identified patient information and baseline findings

**Figure 1:**
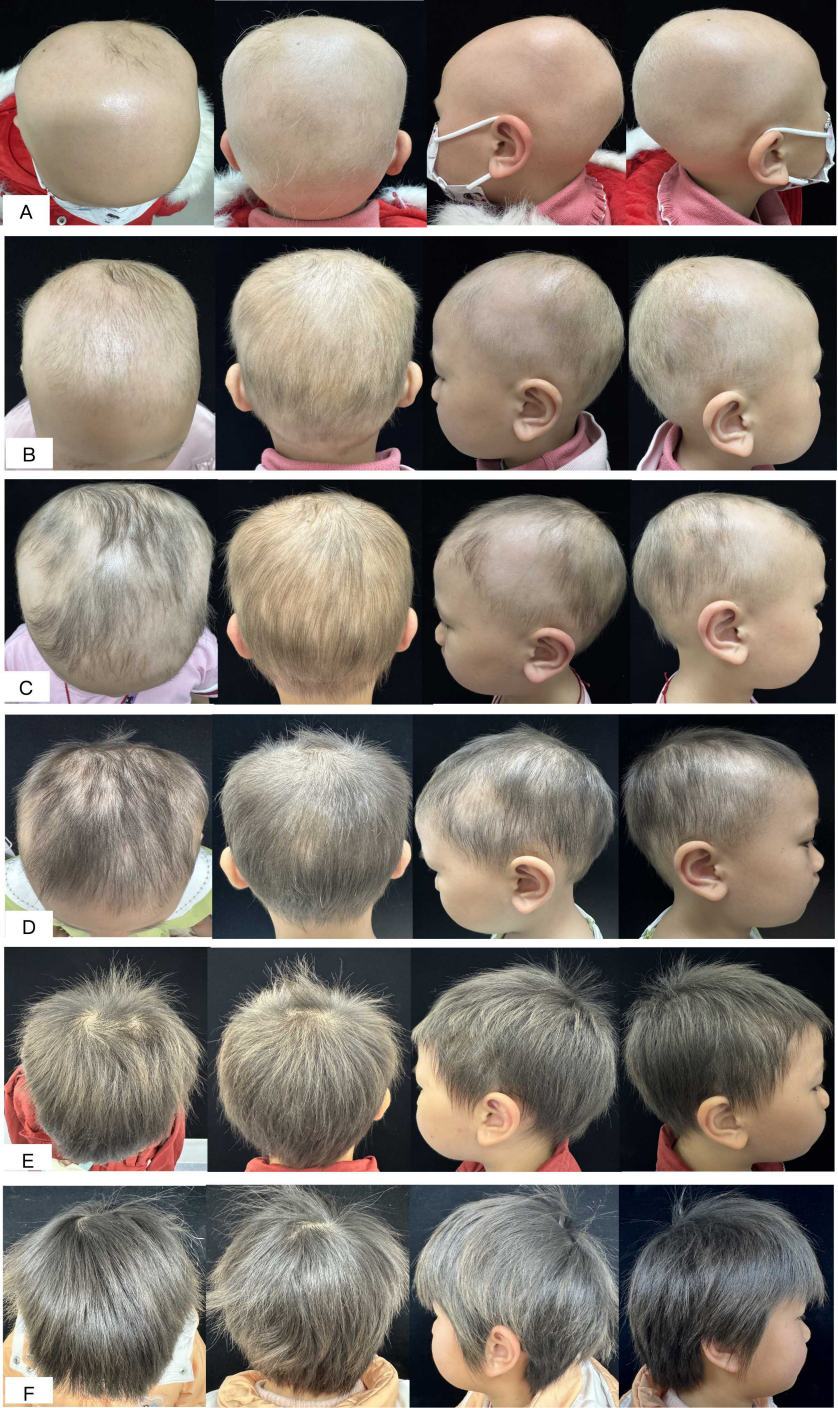
Serial clinical photographs of the scalp during dupilumab treatment. **(A)** Baseline. **(B)** Week 8. **(C)** Week 16. **(D)** Week 24. **(E)** Week 30. **(F)** Week 48.

A 7-year-old girl presented with extensive scalp hair loss of more than 2 years’ duration. She had a history of atopic dermatitis, no other known autoimmune disease, and no relevant family history. Physical examination showed extensive patchy scalp alopecia with a Severity of Alopecia Tool (SALT) score of 95, without eyebrow or eyelash involvement (**Figure 1A**). Concurrent mild-to-moderate atopic dermatitis was noted, presenting as scattered erythema, scaling, and excoriations on both upper limbs, without papules or exudation. The Dermatology Life Quality Index (DLQI) score was 24, although this instrument has not been specifically validated for pediatric populations. Overall, the clinical findings were consistent with a diagnosis of atopic dermatitis.

### Diagnostic assessment

**Figure 2:**
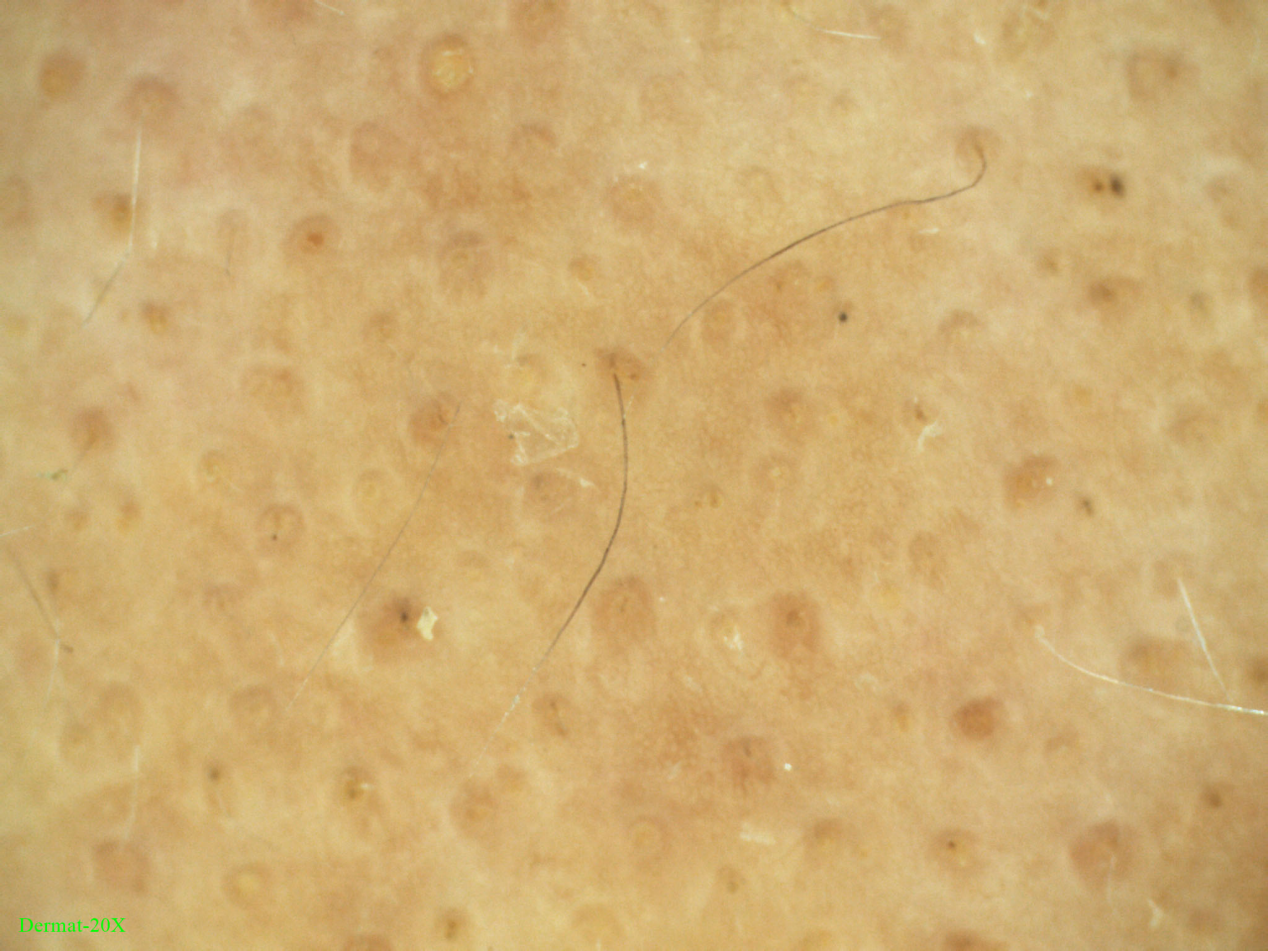
Baseline dermoscopic findings of the alopecic scalp, showing black dots, yellow dots, and a small number of vellus hairs.

Laboratory investigations showed a serum total Immunoglobulin E (IgE) level of 2300 IU/mL. Complete blood count, liver and kidney function, electrolytes, blood lipids, autoantibodies, and screening for hepatitis, HIV, and Treponema pallidum were all normal or negative. Baseline dermoscopy showed black dots, yellow dots, and a small number of vellus hairs in the alopecic areas (**Figure 2**). Previous treatments included oral methylprednisolone (4 mg/day for 1 month), intralesional triamcinolone acetonide injections (5 mg/mL every 6 weeks for 2 sessions), and topical corticosteroids.Improvement had been observed during treatment, but rapid relapse occurred after dose reduction or withdrawal. Improvement had been observed during treatment, but rapid relapse occurred after dose reduction or withdrawal.

### Therapeutic intervention

Given the refractory course of her severe alopecia areata and the coexistence of mild-to-moderate atopic dermatitis, dupilumab was initiated off label primarily for the treatment of AA after a thorough discussion with the patient’s guardians and the acquisition of informed consent. Dupilumab was initiated at a dose of 300 mg subcutaneously every 4 weeks. At baseline, this was combined with topical corticosteroids for AD. After clear improvement by week 16, topical corticosteroids were discontinued and 2% topical minoxidil once daily was added. When the SALT score had decreased to 5 at week 30, the dupilumab dosing interval was successfully extended to every 6 weeks.

### Follow-up and outcomes

**Figure 3:**
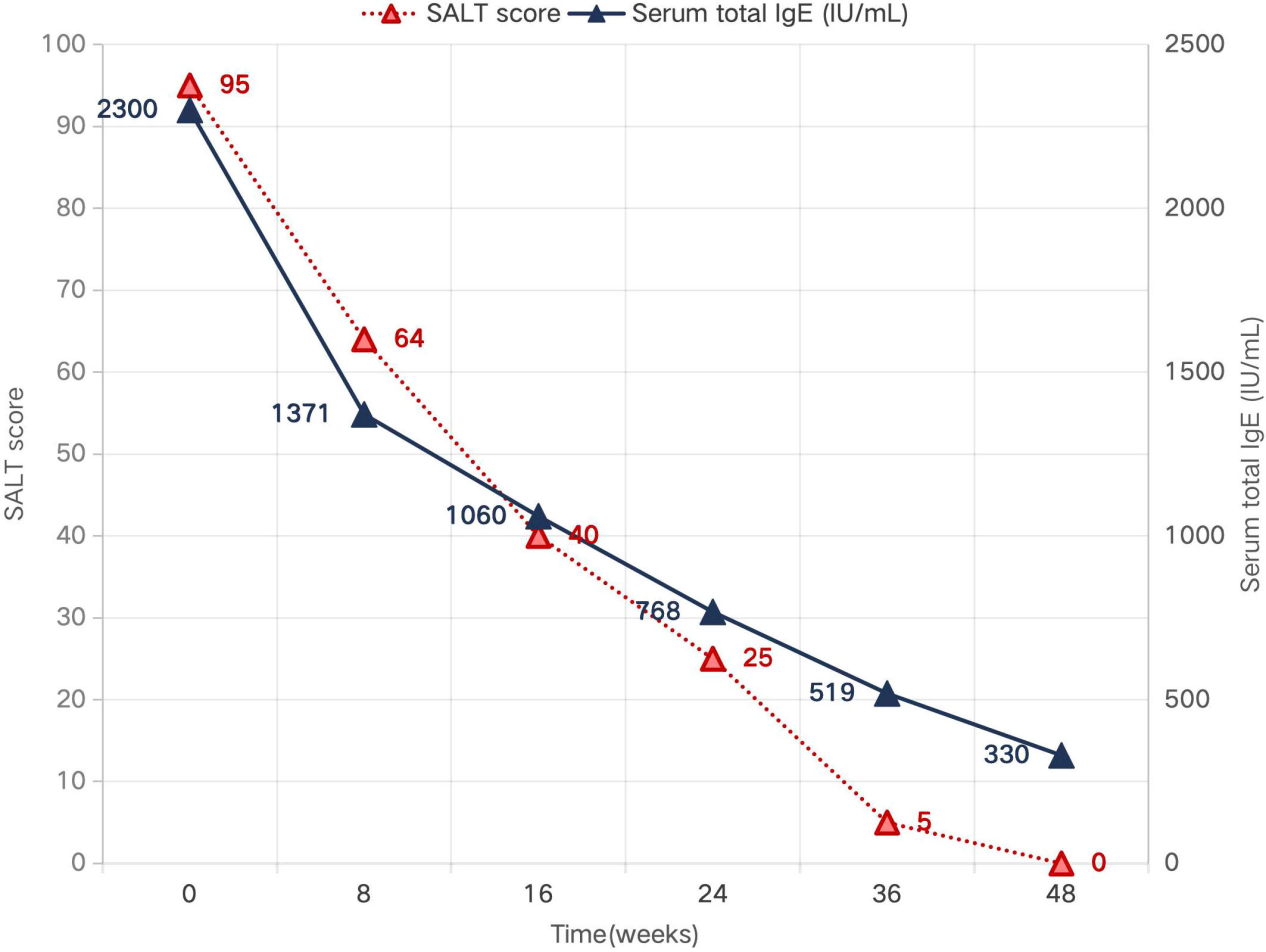
Dynamic changes in SALT score and serum total IgE level during treatment from baseline to week 48.

The patient exhibited a progressive response to treatment. By week 8, sparse hair regrowth was observed, and her atopic dermatitis had improved markedly, with substantial resolution of erythema on the upper limbs. By week 16, hair regrowth had continued, prompting the discontinuation of topical corticosteroids and the initiation of topical minoxidil (as detailed in **Table 1**). By week 24, the alopecic area had further reduced, and her atopic dermatitis had cleared completely with full resolution of pruritus. At week 30, a near-complete response was achieved (SALT 5), and the dupilumab dosing interval was extended to every 6 weeks. Finally, at week 48, complete remission was confirmed with a fully covered scalp (SALT 0) (**Figure 1F**), accompanied by a parallel decline in serum total IgE to 330 IU/mL (**Figure 3**).

**Table 1:** Timeline of treatment and clinical outcomes.

Time point	Clinical findings	Treatment/adjustment
Baseline	Extensive scalp alopecia; SALT 95; IgE 2300 IU/mL; Atopic dermatitis (AD) present (**Figure 1A**).	Dupilumab 300 mg every 4 weeks + 0.05%halometasone cream every other day + Compound Flumetasone Ointment every other day
Week 8	Sparse hair regrowth; SALT 64; AD improved (**Figure 1B**).	Continued same regimen.
Week 16	Further hair regrowth, residual bilateral frontotemporal alopecia; SALT 40 (**Figure 1C**).	Topical corticosteroids discontinued; 2% topical minoxidil added once daily.
Week 24	Alopecic area further reduced; SALT 25; pruritus markedly relieved (**Figure 1D**).	Continued dupilumab + minoxidil.
Week 30	Near-complete response; SALT 5 (**Figure 1E**).	Dupilumab interval extended to every 6 weeks.
Week 48	Complete remission; SALT 0; scalp fully covered with hair; IgE 330 IU/mL (**Figure 1F**, **Figure 3**).	Maintained on dupilumab every 6 weeks.

After the first dupilumab injection, the patient developed transient edematous wheals on both upper eyelids and upper limbs. The reaction resolved rapidly after oral loratadine, and no similar event occurred after subsequent injections. No serious adverse event was observed during follow-up.

## Discussion

The treatment of severe alopecia areata in children remains challenging, and conventional therapies often provide limited benefit with notable adverse effects (5–8). In recent years, the role of the Th2 pathway in alopecia areata has become clearer, providing a rationale for targeted treatment (9, 10). A phase IIa clinical trial confirmed the efficacy of dupilumab in adults with alopecia areata and proposed baseline IgE ≥200 IU/mL as a predictor of treatment response (12). Subsequent and prior case reports have also suggested that dupilumab can be effective and well tolerated in both adults (13, 14) and children (15–17) with AA and concomitant atopic dermatitis. However, detailed data remain limited for children with extremely high baseline IgE levels, particularly regarding long-term efficacy, dynamic IgE change, and optimization of maintenance dosing.

In the present case, the baseline IgE level was 2300 IU/mL, far above the response-associated threshold proposed by Guttman-Yassky et al. (12), and complete remission was achieved within 48 weeks. This observation supports the hypothesis that marked Th2 skewing may identify a subgroup of patients particularly likely to benefit from IL-4/IL-13 blockade. The outcome is consistent with the favorable responses seen in high-IgE pediatric patients reported by Huang et al. (14), while also complementing the report by Cai et al. (13), in which a younger child with normal IgE responded well, suggesting that elevated IgE may enrich for responders without being an absolute prerequisite (18, 19).

It is important to note, however, that the response to dupilumab in alopecia areata is not universal, and the underlying immunological interplay is complex. Cases of non-response, including in pediatric patients with concomitant atopic dermatitis, have been reported (20). Moreover, paradoxical new-onset AA or worsening of pre-existing AA during dupilumab therapy for AD has also been described, underscoring the heterogeneity of AA pathogenesis (21). The remarkable response observed in our patient, in contrast to these variable outcomes, may be related to her distinct immunophenotype, characterized by markedly elevated serum IgE levels, strongly suggestive of predominant Th2-driven inflammation. This observation supports the possibility of a “Th2-high/IgE-high” endotype in AA that may be particularly responsive to IL-4/IL-13 blockade. In our case, the parallel decline in serum IgE levels and SALT score provides additional serological support for this hypothesis.

Hair regrowth appeared by week 8 in our patient, which was somewhat faster than the average time to response summarized by Metko et al. (16). In contrast, Wei et al. (15) described a slower and less favorable early course in a 5-year-old boy with ophiasis-pattern alopecia areata and a substantially lower IgE level. Although no firm causal conclusion can be drawn from a single case, the present outcome raises the possibility that very high baseline IgE may correlate not only with response probability but also with response speed. Another notable feature was the parallel decline in IgE and SALT score, which provides practical serologic support for the biologic plausibility of dupilumab in this setting and suggests that dynamic IgE monitoring may serve as an auxiliary indicator during follow-up.

A further strength of this case is the maintenance strategy. Most published reports have used dosing intervals of 2–4 weeks, but there is still no consensus on how to individualize maintenance therapy once a substantial response has been achieved. In our patient, extension of the interval to every 6 weeks after the SALT score had decreased to 5 was followed by maintained efficacy through week 48. This suggests that moderate interval extension, rather than abrupt discontinuation, may be a practical strategy to reduce treatment burden and cost while preserving disease control in selected patients.

This report has several limitations. It describes a single patient, and dupilumab was used together with topical corticosteroids early in the course and topical minoxidil later, so the independent contribution of each adjunctive therapy cannot be quantified. In addition, scalp biopsy and cytokine profiling were not performed. Even so, the clear temporal association between dupilumab treatment, progressive hair regrowth, declining IgE levels, and successful interval extension provides clinically relevant evidence for the role of Th2-targeted therapy in a subset of pediatric alopecia areata.

## Conclusion

This case shows that dupilumab can be associated with rapid and sustained complete remission in a child with severe alopecia areata and markedly elevated baseline IgE. It further supports baseline IgE as a potentially useful biomarker for treatment selection, suggests that serial IgE measurement may help track response, and provides a practical example of successful extension of the dosing interval during maintenance therapy.

## Patient perspective

The patient’s mother reported that hair loss had caused substantial emotional distress, school-related embarrassment, and prolonged treatment burden for the family. She described dupilumab as the first therapy associated with clear and sustained regrowth, noted that her daughter gradually regained confidence as the scalp hair returned, and considered interval extension from 4 weeks to 6 weeks helpful because it reduced the number of injections and overall family burden.

## Data Availability

The raw data supporting the conclusions of this article will be made available by the authors, without undue reservation.
